# Preliminary Evaluation of the Safety and Immunogenicity of a Novel Protein-Based Pneumococcal Vaccine in Healthy Adults Aged 18–49: A Phase Ia Randomized, Double Blind, Placebo-Controlled Clinical Study

**DOI:** 10.3390/vaccines12080827

**Published:** 2024-07-23

**Authors:** Yanxia Wang, Gang Shi, Xue Wang, Zhiqiang Xie, Jinbo Gou, Lili Huang, Haitao Huang, Wangyang You, Ruijie Wang, Yongli Yang, Feiyu Wang, Tao Zhu, Dongyang Zhao

**Affiliations:** 1 Henan Center for Disease Control and Prevention, Zhengzhou 450016, China; wangyanxia99@163.com (Y.W.); xiezqshang@163.com (Z.X.); 13643826177@163.com (L.H.); dsrt12345@163.com (W.Y.); 2 National Institutes for Food and Drug Control, Beijing 100050, China; shigang@nifdc.org.cn; 3 CanSino Biologics Inc., Tianjin 300457, China; xue.wang@cansinotech.com (X.W.); jinbo.gou@cansinotech.com (J.G.); haitao.huang@cansinotech.com (H.H.); ruijie.wang@cansinotech.com (R.W.); feiyu.wang@cansinotech.com (F.W.); 4 Department of Epidemiology and Health Statistics, College of Public Health, Zhengzhou University, Zhengzhou 450001, China; ylyang377@zzu.edu.cn

**Keywords:** protein-based pneumococcal vaccine, pneumococcal surface protein A, pneumolysin protein, immunogenicity

## Abstract

**Background**: Protein-based pneumococcal vaccines (PBPVs) may offer expanded protection against *Streptococcus pneumoniae* and tackle the antimicrobial resistance crisis in pneumococcal infections. This study examined the safety and immunogenicity in healthy adults vaccinated with three doses of a protein-based pneumococcal vaccine containing pneumococcal surface protein A (PspA) (PRX1, P3296 and P5668) and in combination with a recombinant detoxified pneumolysin protein (PlyLD). **Methods**: This phase Ia randomized, double blind, placebo-controlled clinical study enrolled healthy adults aged 18–49 years. The participants were randomized into experimental (low-dose, medium-dose, high-dose) and placebo groups in a ratio of 3:1. Three doses of investigational vaccine were given to the participants with an interval of two months. Safety endpoints included the occurrence of total adverse reactions, solicited local and systemic adverse reactions, unsolicited adverse reactions, serious adverse events (SAEs), and several laboratory parameters. Immunogenicity endpoints included geometric mean titers (GMT) of anti-PspA (PRX1, P3296 and P5668) and anti-PlyLD antibodies level as determined by ELISA, seropositivity rates of PspA and PlyLD antibodies (>4-fold increase) and neutralization activity of anti-Ply antibody in serum. **Results**: A total of 118 participants completed the study of three doses. The candidate PBPV was safe and well-tolerated in all experimental groups. No vaccine-related SAEs were observed in this study. Most solicited adverse reactions were mild and transient. The most frequently reported solicited adverse reactions in the medium- and high-dose groups was pain at the injection site, while in the low-dose group it was elevated blood pressure. The immunogenicity data showed a sharp increase in the GMT level of anti-PspA-RX1, anti-PspA-3296, anti-PspA-5668, and anti-PlyLD antibodies in serum. The results also showed that the elicited antibodies were dosage-dependent. The high-dose group showed a higher immune response against PspA-RX1, PspA-3296, PspA-5668, and PlyLD antigens. However, repeat vaccination did not increase the level of anti-PspA antibodies but the level of anti-PlyLD antibody. High seropositivity rates were also observed for anti-PspA-RX1, anti-PspA-3296, anti-PspA-5668, and anti-PlyLD antibodies. In addition, a significant difference in the GMT levels of anti-Ply antibody between the high-, medium-, and low-dose groups post each vaccination were indicated by neutralization activity tests. **Conclusions**: The PBPV showed a safe and immunogenic profile in this clinical trial. Taking into consideration both safety and immunogenicity data, we propose a single dose of 50 µg (medium dose) of PBPV as the optimum approach in providing expanded protection against *Streptococcus pneumoniae*.

## 1. Introduction

*Streptococcus pneumoniae* (*S. pneumoniae*), also known as pneumococcus, is a Gram-positive bacterium that inhabits the mucosal surfaces of the human upper respiratory tract (URT) and is responsible for a wide range of diseases, including pneumonia, otitis media, meningitis, and sepsis [1,2]. The polysaccharide capsule surrounding its cell wall is a crucial virulence factor. Currently, 101 different serotypes of pneumococcal capsular polysaccharides have been identified [3]. Due to increasing resistance to penicillin and other antibiotics, *S. pneumoniae* was classified by the WHO in 2017 as one of the ‘priority pathogens’ requiring urgent development of new antibiotics [4], underscoring the need for new vaccine strategies for prevention [5].

Two pneumococcal vaccines, namely the pneumococcal polysaccharide vaccine (PPSV) and the pneumococcal conjugate vaccine (PCV), have been employed to target the predominant serotypes responsible for invasive pneumococcal disease (IPD). These vaccines have significantly decreased the incidence of IPD and pneumococcal pneumonia [6,7,8]. Some studies have shown that, however, PCV vaccination was not able to reduce pneumococcal carriage rates due to the vaccine escape of nonvaccine-type (NVT) strains, especially serotype 3 [9]. At the same time, while PPSV protects against various serotypes, it fails to induce memory B cells or generate isotype switching as it is a T-cell immunogen, which leads to temporary protection, and is only suitable for the elderly population [10]. Moreover, geographical differences in serotype distribution have diminished the effectiveness of these vaccines due to inadequate serotype coverage [11].

Currently, multivalent protein-based pneumococcal vaccines (PBPVs), which potentially induce both mucosal and systemic immunity, provide broad serotype coverage, and prevent IPD, are alternatives to PPSV and PCVs [12]. These vaccine candidates include PnuBioVax, developed through mutation of pneumolysin (Ply), pneumococcal surface protein A (PspA), and pilus-1 subunits [13]; PPrV, a trivalent protein vaccine composed of recombinant proteins PcpA, PlyD1, and pneumococcal histidine triad protein D (PhtD) [14]; and polysaccharide conjugates combining 10 pneumococcal serotypes with pneumolysin toxoid (dPly) and PhtD (PHiD-CV/dPly/PhtD-30) [15].

Additionally, there are three recombinant avirulent *Salmonella Typhi* (RASV) strains, each expressing PspA, and pneumococcal protein-based formulations consisting of Ply, PhtD, and 10-valent pneumococcal non-typeable *Haemophilus influenza* protein D conjugate vaccine (PHiD-CV) (either administered alone or in combination) [16,17]. These candidate vaccines were in various phases of clinical trials and have shown acceptable safety, tolerability, reactogenicity, and immunogenicity profiles.

Several pneumococcal proteins such as PspA, pneumococcal surface protein C (PspC), pneumococcal surface adhesin A (PsaA), PhtD, and pneumococcal hemolysin (pneumolysin, Ply) are currently the most prominent candidate antigens, and have been comprehensively reviewed in numerous studies. These antigens have demonstrated good safety and immunogenicity in animal experiments and/or clinical trials and can protect against pneumococcal infection to varying degrees [18,19,20,21,22,23]. Among these candidate antigens, we are particularly interested in the PspA and Ply proteins as our primary pneumococcal vaccine targets based on promising pre-clinical and clinical findings. For instance, PnuBioVax was developed by modifying the pneumolysin of the *S. pneumoniae* serotype 4 TIGR4 strain to a non-toxic variant, which effectively stimulates CD4 T cell migration, activates the complement system, and toll-like receptor 4 [24,25]. In a preclinical study, rabbit sera immunized with PnuBioVax demonstrated opsonophagocytic killing activity against the vaccine strain TIGR4, as well as serotype strains 6B, 19F, and 15B. Furthermore, incubation of various pneumococcal strains with immunized sera resulted in inhibited pneumolysin-mediated erythrocyte lysis, bacterial agglutination, and reduced invasion of lung epithelial cells in vitro [13,26]. Moreover, PnuBioVax demonstrated safety and immunogenicity in healthy adults aged 18–40 years during a Phase 1 clinical trial conducted by ImmunoBiology Limited (UK) [13]. In contrast, various in vivo studies have indicated that the PspA vaccine can induce cross-protection in animal models against multiple serotypes responsible for invasive diseases and carriage [27,28,29,30,31]. Moreover, a Phase I trial confirmed the immunogenicity of recombinant PspA in humans. Evaluation of serum samples collected before and after immunization in this clinical trial revealed that human antibodies against PspA conferred protection against pneumococcal infection in mice [27].

In this Phase I study, our objective is to evaluate the safety and immunogenicity of a novel PBPV containing PspA proteins originating from different families (PRX1, P3296, and P5668) in combination with a genetically detoxified PLY-derivative (PlyLD) in healthy adults aged 18–49 years. Our in-house in vivo studies have confirmed that administration of the candidate vaccine in different animal models was safe and immunogenic, with no evidence of pathological manifestations.

## 2. Methodology

### 2.1. Study Design

A Phase Ia, randomized, double-blind, placebo controlled clinical study was conducted between April 2020 and May 2021 at the Henan Center for Disease Control and Prevention, China. The primary objective of this study was to evaluate the safety of the investigational product in healthy adults, and the secondary objective was to assess the immunogenicity of the investigational vaccine. The study protocol was approved by the Henan Center for Disease Control and Prevention, China. The study was conducted in accordance with the demand of National Medical Products Administration (NMPA), China, the Declaration of Helsinki and Good Clinical Practice. Informed consent was obtained from each participant prior to enrolment.

### 2.2. Participants and Vaccine

Participants were recruited and randomly assigned into low-, medium-, and high-dose groups using Stata16.0 software. In each dosing group, participants were arranged in an experimental group and a placebo group with the ratio of 3:1. Participants were enrolled in an ascending order of dosage, with each stage determined by a safety review. Safety reviews included a set of procedures such as height, weight, body temperature measurement, skin inspections, and electrocardiogram (ECG) analysis. Women of childbearing potential underwent urine pregnancy tests. Examinations of laryngopharyngeal, cardiovascular, and blood pressure functions were conducted. Participants with a medical history of severe illnesses, infectious diseases, and organ excision were excluded.

Eligible participants were healthy adults aged 18–49 years. The exclusion criteria for the first dose included high blood pressure uncontrolled by medication (systolic ≥ 140 mmHg, diasytolic ≥ 90 mmHg); pregnant or planning to become pregnant; breastfeeding; a history of pneumonia or complications from invasive pneumococcal disease within 3 years before vaccination; allergic conditions or a history of allergic reactions to any vaccine products; axillary temperature > 37 °C before vaccination; and any other reasons deemed appropriate by the investigators. The exclusion criteria for receiving the second and third doses were similar, with additional considerations for severe allergic or adverse events related to the first vaccination.

The active ingredients of PBPV were 50 μg each of PspA RX1, PspA3296, PspA 5668 and PlyLD. The recombinant proteins were fermented, purified, mixed, and prepared by adding aluminium hydroxide adjuvant for adsorption. The vaccine production complied with the GMP standard [32]. This vaccine was administered intramuscularly as a 0.5 mL injection into the deltoid muscle of the non-dominant arm. The participants received three doses of the investigational vaccine at month 0, 2, and 4. The low-dose group received 0.2 mL (20 μg of PBPV), the medium-dose group 0.5 mL (50 μg of PBPV), and the high-dose group 1.0 mL (100 μg of PBPV).

### 2.3. Safety Assessment

Within 30 days following vaccination, both solicited and unsolicited adverse reactions were documented for each study group. The severity of adverse reactions was categorized as mild (grade 1), moderate (grade 2), severe (grade 3), or potentially life-threatening (grade 4). Throughout the 180-day study period, serious adverse events (SAEs) were monitored, defined as any medical occurrence during the trial that was life-threatening, resulted in death, necessitated hospitalization or prolonged existing hospitalization, impacted work productivity, or involved any birth defect or congenital anomaly.

### 2.4. Immunogenicity Assessment

Blood samples were collected for immunogenicity assessments before each dose, and at day 30 after each dose for each group. Blood was centrifuged at 3000 rpm for 10–15 min and aliquoted into serum. Samples were then stored at −20 °C until analysis. Antibody levels were quantified to measure the geometric mean titers (GMTs) and seropositivity using ELISA coated with PspA-RX1, PspA-3296, PspA-5668, and PlyLD proteins with assay cut-offs on day 30 post vaccination. The serum samples before and after immunization were diluted 100-fold with 7–10 gradients of multiplicative dilution. The steps of sample addition—secondary antibody incubation—color development—termination were followed, and finally the OD values were measured at 450/630 nm. These cut-offs were in accordance with lower limit of quantification. Participants with antibody levels below these technical cut-offs were regarded as antibody negative, although this did not necessarily indicate true negatives as these are not a clinical cut-off. Seropositivity was defined as four-fold increase in the antibody levels of PspA-RX1, PspA-3296, PspA-5668, and PlyLD proteins before and post vaccination. A Ply neutralizing test assay was used to measure the neutralizing activity of Ply at day 30 post each vaccination. The serum samples were inactivated during sample processing, and then were diluted with saline as diluent for n-fold starting dilution and two-fold gradient dilution. Haemolysis inhibition of 100% (100 µL saline), haemolysis inhibition of 0% (100 µL purified water), and Ply haemolysis control (50 µL saline, 50 µL Ply) were set as control. For Ply neutralization reaction, wild-type Ply were diluted to a defined concentration and diluted serum (all wells except controls) was added. Subsequently, 2% rabbit erythrocytes were added to all wells, 100 µL/well. Mix well and put into a 37 °C constant temperature incubator role for 60 min. Centrifuge at 1300× *g* for 10 min after the action, take 100 µL of the supernatant and add it into the corresponding wells of a new 96-well microtiter plate, and measure the OD450 nm value.

### 2.5. Statistical Analysis

The Chi-squared test was used to analyze the distribution of gender and age between the low-, medium-, and high-dose groups. AEs were coded using the Medical Dictionary for Regulatory Activities (MedDRA) version 24.0. The incidence of Treatment Emergent Adverse Events (TEAEs) and vaccine-related TEAEs was summarized by organ system, preferred term, severity, and relationship to the investigational product. ARs were considered as vaccine-related AEs. Abnormal laboratory safety and vital signs data were tabulated. Absolute and changes from baseline haematology, biochemistry, and vital signs parameters were summarized descriptively. For safety assessment, the incidence of solicited and unsolicited adverse events between different dosage groups was calculated with exact 95% confidence intervals (CIs) using Fisher’s exact test or the Chi-squared test. For immunogenicity assessment, the analysis was based on Per Protocol Set (PPS). The PPS is a subset of the Full Analysis Data Set (FAS), and includes data from participants fully compliant with the protocol. This approach ensures the data reflect the therapeutic effects according to the scientific model. In this study, GMTs and seropositivity rates of different experimental and placebo groups were calculated with 95% CIs using Kruskal–Wallis or Chi-squared tests. Analyses were performed using Statistical Analysis System (SAS) version 9.4 (70278724).

## 3. Results

### 3.1. Study Participants and Demographics

**Figure 1:**
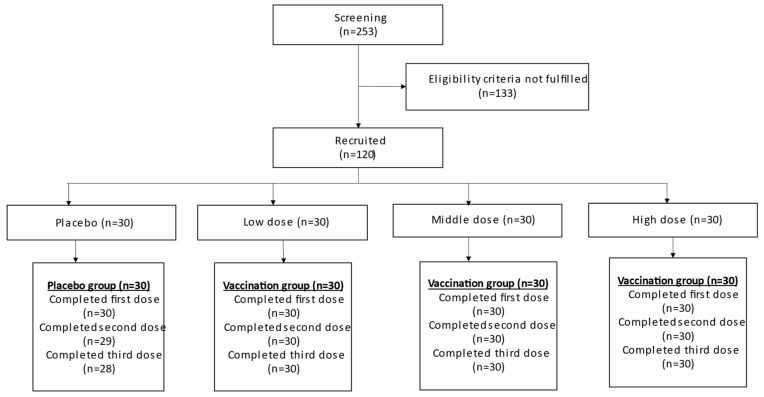
Participant flow diagram.

A total of 253 participants were screened in this study, with 120 participants eligibly enrolled and 118 completing the study. Two participants from the placebo group withdrew during the second and third doses of vaccination. All participants from the low-, medium-, and high-dose experimental groups completed all three vaccinations. The details are illustrated in Figure 1.

Demographic characteristics of the participants are shown in Table 1. Of the 120 participants, 57 were male and 63 were female. The average ages in the placebo, low-dose, medium-dose, and high-dose groups were 37.90, 40.57, 36.08, and 36.77 years, respectively. There was a statistically significance between the genders (*p* = 0.002) and mean ages (*p* = 0.046) of the four groups.

### 3.2. Safety Evaluation of PBPV Vaccine in Healthy Adults

As shown in Table 2, within 30 days after the last vaccination, the incidence of total adverse reactions in the placebo, low-dose, medium-dose, and high-dose groups was 46.67%, 76.67%, 63.33%, and 90.00%, respectively. The incidence of local adverse reactions was 13.33%, 53.33%, 60.00%, and 83.33%, respectively. The medium-dose group had a significantly lower rate of local adverse reactions compared to the placebo group (*p* < 0.001), while no significant difference was observed in the incidence of systemic adverse reactions. Within 30 days after three doses of vaccination, the incidence of other adverse reactions in the placebo, low-dose, medium-dose, and high-dose groups was 20.00%, 30.00%, 10.00%, and 3.33%, respectively, with no significant difference between the medium-dose group and the placebo group. The incidence of pain at the injection site in the placebo, low-dose, medium-dose, and high-dose groups was 13.33%, 53.33%, 60.00%, and 83.33% within 30 days after three doses of vaccination. Compared with the placebo group, the medium-dose group had significantly higher incidence of pain at the injection site (*p* < 0.001). The systemic adverse reactions in all groups within 30 days post three doses of vaccination were mainly fever and fatigue. The incidence of fever in the placebo, low-dose, medium-dose, and high-dose groups was 13.33%, 6.67%, 13.33%, and 20.00%, respectively, with no statistically significance when compared between the medium-dose group and the placebo group (*p* > 0.999). There were only two cases of fatigue in the placebo group and two cases in the high-dose group. Noticeably, there was a high incidence of abnormal systolic and diastolic blood pressure in the low-dose group. However, no significant difference was found between the placebo group and the medium-dose group (*p* > 0.999). No vaccine-related SAE was reported in the study.

**Table 2:** Overview of adverse reactions within 30 days after complete immunization (SS).

	Control Group	Experimental Groups			*p* Value *
	Placebo (n = 30)	Low-Dose (n = 30)	Medium-Dose (n = 30)	High-Dose (n = 30)	
All adverse reactions	14 (46.67%)	23 (76.67%)	19 (63.33%)	27 (90.00%)	0.194
Local adverse reactions	4 (13.33%)	16 (53.33%)	18 (60.00%)	25 (83.33%)	<0.001
Systemic adverse reactions	8 (26.67%)	4 (13.33%)	4 (13.33%)	8 (26.67%)	0.197
Other adverse reactions	6 (20.00%)	9 (30.00%)	3 (10.00%)	1 (3.33%)	0.470
Erythema	0 (0.00%)	1 (3.33%)	1 (3.33%)	1 (3.33%)	>0.999
Pain	4 (13.33%)	16 (53.33%)	18 (60.00%)	25 (83.33%)	<0.001
Swelling	0 (0.00%)	1 (3.33%)	4 (13.33%)	8 (26.67%)	0.121
Itchiness	0 (0.00%)	0 (0.00%)	1 (3.33%)	0 (0.00%)	>0.999
Induration	0 (0.00%)	0 (0.00%)	1 (3.33%)	0 (0.00%)	>0.999
Hypersensitivity	1 (3.33%)	0 (0.00%)	0 (0.00%)	0 (0.00%)	>0.999
Nausea	0 (0.00%)	1 (3.33%)	0 (0.00%)	1 (3.33%)	-
Fever	4 (13.33%)	2 (6.67%)	4 (13.33%)	6 (20.00%)	>0.999
Diarrhea	1 (3.33%)	1 (3.33%)	0 (0.00%)	1 (3.33%)	>0.999
Cough	1 (3.33%)	0 (0.00%)	1 (3.33%)	0 (0.00%)	>0.999
Fatigue	2 (6.67%)	0 (0.00%)	0 (0.00%)	2 (6.67%)	0.472
Headache	1 (3.33%)	1 (3.33%)	0 (0.00%)	1 (3.33%)	>0.999
Abnormal white blood cell counts	1 (3.33%)	0 (0.00%)	0 (0.00%)	0 (0.00%)	>0.999
Proteinuria	2 (6.67%)	0 (0.00%)	0 (0.00%)	0 (0.00%)	0.472
Abnormal systolic blood pressure	0 (0.00%)	7 (23.33%)	0 (0.00%)	0 (0.00%)	-
Abnormal diastolic blood pressure	3 (10.00%)	8 (26.67%)	2 (6.67%)	0 (0.00%)	>0.999
Bilirubin abnormality	0 (0.00%)	1 (3.33%)	0 (0.00%)	1 (3.33%)	-
Physical discomfort	0 (0.00%)	0 (0.00%)	1 (3.33%)	0 (0.00%)	>0.999

* Comparison between medium-dose group and placebo group.

**Table 3:** Adverse reactions within 30 days after first doses (SS).

	Control Group	Experimental Groups			*p* Value *
	Placebo (n = 30)	Low-Dose (n = 30)	Medium-Dose (n = 30)	High-Dose (n = 30)	
All adverse reactions	12 (40.00%)	16 (53.33%)	7 (23.33%)	17 (56.67%)	0.165
Local adverse reactions	1 (3.33%)	6 (20.00%)	3 (10.00%)	14 (46.67%)	0.605
Systemic adverse reactions	8 (26.67%)	2 (6.67%)	4 (13.33%)	4 (13.33%)	0.197
Other adverse reactions	6 (20.00%)	9 (30.00%)	2 (6.67%)	1 (3.33%)	0.255
Erythema	0 (0.00%)	0 (0.00%)	1 (3.33%)	1 (3.33%)	>0.999
Pain	1 (3.33%)	6 (20.00%)	2 (6.67%)	13 (43.33%)	>0.999
Swelling	0 (0.00%)	0 (0.00%)	1 (3.33%)	4 (13.33%)	>0.999
Hypersensitivity	1 (3.33%)	0 (0.00%)	0 (0.00%)	0 (0.00%)	>0.999
Nausea	0 (0.00%)	1 (3.33%)	0 (0.00%)	1 (3.33%)	-
Fever	4 (13.33%)	0 (0.00%)	4 (13.33%)	3 (10.00%)	>0.999
Diarrhea	1 (3.33%)	1 (3.33%)	0 (0.00%)	1 (3.33%)	>0.999
Cough	1 (3.33%)	0 (0.00%)	0 (0.00%)	0 (0.00%)	>0.999
Fatigue	2 (6.67%)	0 (0.00%)	0 (0.00%)	1 (3.33%)	0.472
Headache	1 (3.33%)	1 (3.33%)	0 (0.00%)	1 (3.33%)	>0.999
Abnormal white blood cell counts	1 (3.33%)	0 (0.00%)	0 (0.00%)	0 (0.00%)	>0.999
Proteinuria	2 (6.67%)	0 (0.00%)	0 (0.00%)	0 (0.00%)	0.472
Abnormal systolic blood pressure	0 (0.00%)	7 (23.33%)	0 (0.00%)	0 (0.00%)	-
Abnormal diastolic blood pressure	3 (10.00%)	8 (26.67%)	1 (3.33%)	0 (0.00%)	0.605
Bilirubin abnormality	0 (0.00%)	1 (3.33%)	0 (0.00%)	1 (3.33%)	-
Physical discomfort	0 (0.00%)	0 (0.00%)	1 (3.33%)	0 (0.00%)	>0.999

* Comparison between medium-dose group and placebo group.

In addition, Table 3 showed that, within 30 days post first vaccination, the incidence of total adverse reactions was higher in the low-dose and high-dose groups when compared with the medium-dose group. Although no statistical difference in total adverse reactions between the medium-dose group and the low-dose group was observed post complete vaccination, there was a significant association of total adverse reactions with the high-dose group compared with medium-dose group. This indicates that the medium-dose vaccine has a better safety profile than that of the high-dose, and it also suggests better safety than that of the low-dose post first dose of vaccination. Taken together, our study suggests that the safety profile of the medium group is the most favorable among all three dosages.

### 3.3. Immunogenicity of PBPV Vaccine in Health Adults

Since the components of pneumococcal protein vaccines differ from the components of polysaccharide vaccines, the immunogenicity analysis is also different from that of marketed pneumococcal vaccines. Table 4 shows the geometric mean titer (GMT) of anti-PspA-RX1, anti-PspA-3296, anti-PspA-5668, and anti-PlyLD antibodies in serum by ELISA. Compared to the placebo group, all experimental groups (low-dose, medium-dose, and high-dose) induced higher immune responses against PspA-RX1, PspA-3296, PspA-5668, and PlyLD antigens in serum. Before vaccination, the differences in the GMT of PspA-RX1, PspA-3296, and PspA-5668 antibodies were not statistically significant among the three experimental groups. The high-dose group, however, showed a relatively higher immune response post vaccination. In addition, the GMT levels of PspA-RX1, PspA-3296, PspA-5668, and PlyLD antibodies increased over time when compared between pre-dosing and at day 30 post each dosing, indicating its dosage dependence. The GMT levels in the medium-dose group were slightly higher than in the low-dose group, whilst the GMT levels in the high-dose group were the highest among all three. All post-vaccination differences were statistically significant between experimental groups.

Table 5 depicts the seropositivity rates of anti-PspA-RX1, anti-PspA-3296, anti-PspA-5668, and anti-PlyLD antibodies in serum. All experimental groups achieved relatively high seropositivity rates at all time points (the seropositivity rates of anti-PlyLD at day 30 after dose 1 in experimental groups were relatively low). Nonetheless, the seropositivity rates did not differ significantly between each experimental group post each dosing, indicating lack of dosage dependence. In addition, the GMT and seropositivity of anti-PspA-RX1, anti-PspA-3296, and anti-PspA-5668 antibodies in each experimental group (low-dose, medium-dose, and high-dose) did not show a significant increase with repeated vaccination. This suggests that a single vaccination dose is sufficient to stimulate adequate immune responses against PspA-RX1, PspA-3296, and PspA-5668 antigens. Conversely, the GMT levels of anti-PlyLD antibody escalated gradually with repeated vaccination in all experimental groups. Overall, our study suggests that the high dose is the most immunogenic compared to the low and medium doses, while the immunogenicity levels in the medium- and low-dose groups were similar.

The neutralization test activity of anti-Ply antibody in serum is shown in Table 6. Our results showed that the GMT levels of anti-Ply antibody significantly differed between the high-, middle- and low-dose groups at day 30 post each vaccination. The GMT levels of anti-Ply antibody in the high-dose group were relatively higher than the middle- and low-dose groups, while the GMT levels of Ply neutralization antibody in the middle- and low-dose groups were almost similar. Notably, at day 30 post each dosing, the percentage of neutralization test activity of anti-Ply antibody ≥ 4 was higher in the middle-dose group when compared to the low- and high-dose groups, although the differences were not statistically significant. Additionally, the percentage of neutralization test activity of anti-Ply antibody ≥ 4 at day 30 (post dose 2 and 3 of vaccination) was higher in all experimental groups when compared to day 30 post dose 1 (Table 7).

## 4. Discussion

Pneumococcal surface protein A (PspA) is among the extensively studied candidate proteins for combatting pneumococcal infections, addressing limitations of polysaccharide-based vaccines. This cell-wall-associated protein is a critical virulence factor expressed by nearly all strains, displaying variation and categorized into three families and six clades: Family 1 (clades 1 and 2), Family 2 (clades 3, 4, and 5), and Family 3 (clade 6) [33,34]. More than 90% of strains express PspA from Family 1 or Family 2 [35,36]. Combining PspA antigens from different families, such as Family 1 (clade 2) and Family 2 (clades 3 and 4), is proposed to provide broad protection against diverse *S. pneumoniae* isolates [37,38]. Furthermore, PspA’s polymorphism undergoes immunological selection, confirming its accessibility to antibodies on pneumococcal surfaces [39]. Studies have shown that immunization with recombinant PspA from strain Rx1 (Family 1, clade 2) induces extensive, cross-reactive antibody responses in healthy adults [40]. However, several concerns have been raised about using protein-based candidate vaccines against pneumococcal infections, such as chemical instability, variability in sequence and expression level, and potential reactogenicity, as well as autoimmune properties [23].

To our knowledge, this is the first clinical trial to evaluate the safety and immunogenicity of a combination of PspA and Ply proteins in healthy human participants. In this study, three PspA molecules (PRX1, P3296 and P5668) and a genetically engineered pneumolysin protein (PlyLD) were selected to formulate a recombinant pneumococcal protein-based vaccine. The RX 1 protein belongs to family 1 (subclass 2), 3296 protein belongs to family 2 (subclass 30), and 5668 proteins belong to family 2 (subclass 4). Compared to the current market’s 23-valent pneumonia polysaccharide vaccine and the 13-valent conjugate vaccine currently on the market, this formulation is expected to have a higher coverage rate of above 94% of the population. High coverage rates could effectively prevent serotype substitution and emergence of antibiotic-resistant pneumococcus. Additionally, protection against *S. pneumoniae* would not be limited by the serotype of *S. pneumoniae*, especially for protein vaccines which utilize surface proteins or toxin proteins shared by *S. pneumoniae* as antigens. The vaccine also induces T cell-dependent immune responses and is thus immunogenic when used in infants and the elderly by generating immune memory [23]. Moreover, protein vaccines generated through gene recombination technology offer simplicity, low cost, scalability, and easy quality control, prompting their global use.

Our candidate PBPV vaccine, comprising PspA-RX1, PspA-3296, PspA-5668, and PlyLD, was determined to be safe and well-tolerated in healthy adults who received intramuscular injections of three doses (20, 50, or 100 µg). Local reactions tended to be more frequent and severe with higher doses, particularly in the high-dose group. However, no significant adverse events typically associated with vaccines, such as fatigue, anorexia, diarrhea, nausea/vomiting, fever, or non-injection site myalgia were reported in any of the experimental groups. There were no notable increases in solicited or unsolicited adverse events with increasing doses or repeated administrations at two-month intervals. These findings suggest that the safety profile supports further clinical investigation of this PBPV vaccine in target populations. Similarly, other clinical trials investigating protein-based candidate vaccines have also demonstrated good safety and tolerability in adults [16,41,42]. Also, we found that the medium-dose group exhibited a better safety profile than the low- and high-dose groups, reflected by its lower total adverse reaction rate and milder adverse events. Hence, immunization with PBPV vaccine at medium dose may be the optimum for healthy adults.

Utilizing highly conserved pneumococcal proteins as targets for vaccines offers broader and more sustained protection against pneumococcal disease compared to PCVs, with a lower risk of serotype evasion. Two promising antigens for protein-based pneumococcal vaccines include PspA and pneumolysin (Ply). PspA, a choline-binding protein, is particularly promising as a next-generation vaccine target due to its immunogenicity and presence on the surface of nearly all clinical strains of *S. pneumoniae* [43]. This protein is crucial in virulence, affecting the binding of complement C3b and human lactoferrin, thereby disrupting its protective function. Experimental animal models have demonstrated that PspA can protect against various pneumococcal strains [44,45,46]. PspA has shown efficacy in stimulating T cell immune responses and antibody production during invasive pneumococcal disease in adults [37]. Ongoing clinical trials, including a phase I study with a recombinant PspA oral vaccine employing three distinct avirulent strains of *Salmonella typhi* (RASV), each expressing PspA, are investigating its potential. Additionally, research has demonstrated that an oral attenuated RASV expressing PspA provides effective protection against secondary pneumococcal pneumonia in mice [47]. In contrast, Ply is a cholesterol-dependent cytolysin (CDC) present in nearly all *S. pneumoniae* serotypes. Apart from its lytic capabilities, Ply can activate innate and complement immune responses via toll-like receptor-4 (TLR4), LRR-, NOD-, and pyrin domain-containing protein 3 (NLRP3) inflammasomes [25,48,49]. Research has indicated that toxoid derivatives of Ply (dPly) provide substantial protection against pneumococcal infection in experimental animal models [50,51,52,53]. Clinical trials investigating Ply-based vaccines have successfully demonstrated their protective effects and immunogenicity in humans [19,41,54]. Administration of a detoxified PLY derivative to healthy subjects resulted in increased IgG titers against Plytoxoid and enhanced toxin-neutralizing antibody activity [42]. However, the protective efficacy of a pneumococcal vaccine containing PLY toxoid in humans has not been definitively confirmed. Phase II trial results indicated no additional protection against pneumonia, acute otitis media, or infant pneumococcal nasopharyngeal carriage when ply toxoid was included in combinations with other antigens [17,55]. This underscores that Ply toxicity may not fully account for mucosal disease, though its role remains a topic of ongoing debate and it is considered a crucial component in future protein-based pneumococcal vaccines [56].

Our immunogenicity data revealed that a low dose, medium dose, and high dose of the PBPV vaccine elicited significant immune responses. The high-dose group showed a greater advantage than the medium- and low-dose groups due to higher antibody levels, while the medium- and low-dose groups showed a comparable effect since their antibody production was similar. Except for the neutralization activity of anti-Ply antibody in serum, our findings suggested that repeated vaccination has a minimal effect on antibody production as the antibody levels were not significantly increased at day 30 post dose 2 and post dose 3, and at day 60 post dose 3 when compared to the antibody production at day 30 post dose 1. Our study suggests that reducing the number of vaccination doses can also achieve similar antibody levels in healthy adults. Hence, we propose that a single dose of PBPV vaccine could induce appropriate immune responses against pneumococcus in healthy adults.

Given the limited participant pool and absence of established protective antibody thresholds against pneumococcal proteins, our immunogenicity findings should be interpreted cautiously. Moreover, our study exclusively involved healthy adults aged 18–49 years. While these results provide initial insights into the safety, reactogenicity, and immunogenicity prior to potential extension to pediatric populations, they may not generalize across different age groups. Moving forward, our results underscore the need for further development and assessment of this investigational pneumococcal protein-based vaccine in younger age cohorts, who are particularly vulnerable to pneumococcal diseases. Additionally, emerging pneumococcal protein-based vaccines show promise in targeting serotypes not covered by current PCVs.

## 5. Conclusions

This clinical trial examined the safety and immunogenicity of a novel PBPV vaccine at three different dose levels in healthy adults. The immunogenicity and safety results suggest that a single dose of the PBPV vaccine can achieve a substantial level of immunogenicity. Our preliminary data showed that, although the high-dose PBPV vaccine shows a higher level of immunogenicity, the medium dose, with its superior safety profile, may be the optimal immunization strategy. A single dose of 50 µg of the PBPV vaccine could be the most effective approach. We believe this vaccine can prevent serotype replacement scenarios and address the antimicrobial resistance crisis in pneumococcal infections.

## Figures and Tables

**Table 1:** Demographic characteristics of participants.

Group	N	$\bar{X}\pm S$	*p*	Male (%)	Female (%)	*p*
Placebo	30	37.9 ± 7.32	0.046	9 (30.00)	21 (70.00)	0.002
Low-dose	30	40.57 ± 5.27		18 (60.00)	12 (40.00)	
Medium-dose	30	36.08 ± 6.81		9 (30.00)	21 (70.00)	
High-dose	30	36.77 ± 6.52		21 (70.00)	9 (30.00)	
Total	120	-		57 (47.50)	63 (52.50)	

**Table 4:** GMT of Anti-PspA-RX1, Anti-PspA-3296, Anti-PspA-5668, and Anti-PlyLD antibodies by ELISA in control group and experimental groups (PPS).

		Placebo Group	Low-Dose Group	Medium-Dose Group	High-Dose Group	*p* Value *
Anti-PspA-RX1	Pre vaccination	2141.08	2296.56	2564.88	3173.00	0.249
	Post dose 1 (Day 30)	2261.58	20,936.29	21,737.14	44,540.56	0.001
	Post dose 2 (Day 30)	2587.86	27,384.82	29,605.98	47,497.47	0.005
	Post dose 3 (Day 30)	2328.64	25,140.1	28,518.89	44,919.97	0.002
Anti-PspA-3296	Pre vaccination	3095.43	2651.13	3379.49	3663.93	0.147
	Post dose 1 (Day 30)	3208.99	33,385.86	39,313.62	63,364.52	0.003
	Post dose 2 (Day 30)	3522.18	33,617.88	35,487.95	57,259.99	<0.001
	Post dose 3 (Day 30)	3310.01	29,124.23	33,587.17	47,710.40	<0.001
Anti-PspA-5668	Pre vaccination	2230.02	2445.88	3121.36	3265.04	0.232
	Post dose 1 (Day 30)	2420.29	24,846.77	35,722.28	54,934.81	<0.001
	Post dose 2 (Day 30)	2634.07	32,175.2	39,342.8	55,687.68	0.001
	Post dose 3 (Day 30)	2531.17	30,020.91	39,472.51	46,023.05	0.009
Anti-PlyLD	Pre vaccination	3240.87	2602.69	3837.72	4612.39	0.004
	Post dose 1 (Day 30)	3373.41	7666.59	18,252.6	18,710.33	<0.001
	Post dose 2 (Day 30)	3509.42	21,733.08	34,012.33	54,303.20	<0.001
	Post dose 3 (Day 30)	3541.39	21,909.38	42,338.16	52,805.37	<0.001

* The comparison among low-dose, medium-dose, and high-dose groups.

**Table 5:** Seropositivity (>4) of PspA-RX1, PspA-3296, PspA-5668, and PlyLD antibodies by ELISA in control group and experimental groups (PPS).

		Placebo Group	Low-Dose Group	Medium-Dose Group	High-Dose Group	*p* Value *
PspA-RX1	Post dose 1 (Day 30)	0.00	86.70	80.00	90.00	0.654
	Post dose 2 (Day 30)	0.00	93.30	93.30	96.70	>0.999
	Post dose 3 (Day 30)	0.00	93.30	93.30	93.30	>0.999
PspA-3296	Post dose 1 (Day 30)	0.00	90.00	83.30	100.00	0.090
	Post dose 2 (Day 30)	0.00	93.30	90.00	100.00	0.363
	Post dose 3 (Day 30)	0.00	93.30	90.00	100.00	0.363
PspA-5668	Post dose 1 (Day 30)	0.00	90.00	86.70	100.00	0.154
	Post dose 2 (Day 30)	0.00	93.30	100.00	100.00	0.326
	Post dose 3 (Day 30)	0.00	96.70	96.70	100.00	>0.999
PlyLD	Post dose 1 (Day 30)	0.00	36.70	56.70	40.00	0.248
	Post dose 2 (Day 30)	0.00	86.70	93.30	96.70	0.493
	Post dose 3 (Day 30)	0.00	90.00	100.00	96.70	0.318

* Comparison among low-dose, medium-dose, and high-dose groups.

**Table 6:** GMT of anti-Ply antibodies by neutralization test in control group and experimental groups (PPS).

	Placebo Group	Low-Dose Group	Medium-Dose Group	High-Dose Group	*p* Value *
Pre vaccination	7.79	8.74	5.95	20.46	<0.001
Post dose 1 (Day 30)	7.24	15.58	14.11	28.71	0.003
Post dose 2 (Day 30)	8.12	24.06	28.92	64.69	<0.001
Post dose 3 (Day 30)	8.55	27.02	35.70	59.60	<0.001

* Comparison among low-dose, medium-dose, and high-dose groups.

**Table 7:** Seropositivity (>4) of anti-Ply antibodies by neutralization test in control group and experimental groups (PPS).

	Placebo Group	Low-Dose Group	Medium-Dose Group	High-Dose Group	*p* Value *
Post dose 1 (Day 30)	3.60	10.00	30.00	16.70	0.131
Post dose 2 (Day 30)	7.10	36.70	56.70	43.30	0.285
Post dose 3 (Day 30)	17.90	36.70	63.30	36.70	0.057

* Comparison among low-dose, medium-dose, and high-dose groups.

## Data Availability

The data presented in this study are available upon request from the corresponding authors.
